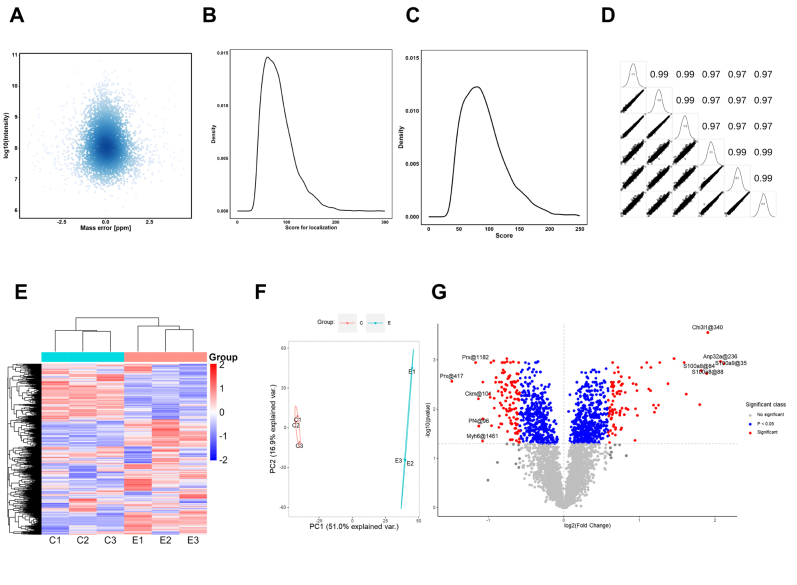# Proteome, Lysine Acetylome, and Succinylome Identify Posttranslational Modification of STAT1 as a Novel Drug Target in Silicosis

**DOI:** 10.1016/j.mcpro.2024.100770

**Published:** 2024-04-17

**Authors:** Tiantian Zhang, Yiyang Wang, Youliang Sun, Meiyue Song, Junling Pang, Mingyao Wang, Zhe Zhang, Peiran Yang, Yiling Chen, Xianmei Qi, Huan Zhou, Zhenzong Han, Yanjiang Xing, Ying Liu, Baicun Li, Jiangfeng Liu, Juntao Yang, Jing Wang

**Affiliations:** 1 State Key Laboratory of Respiratory Health and Multimorbidity, Institute of Basic Medical Sciences Chinese Academy of Medical Sciences, School of Basic Medicine Peking Union Medical College, Beijing, China; 2 Department of Respiratory and Critical Care Medicine, The First Affiliated Hospital of Xi’an Jiaotong University, Xi’an, China; 3 Institute of Basic Medicine, School of Medicine, Tsinghua University, Beijing, China; 4 State Key Laboratory of Common Mechanism Research for Major Disease, Institute of Basic Medical Sciences Chinese Academy of Medical Sciences, School of Basic Medicine Peking Union Medical College, Beijing, China; 5 Department of Respiratory and Critical Care Medicine, Aerospace Center Hospital, Peking University Aerospace School of Clinical Medicine, Beijing, China; 6 Center of Respiratory Medicine, China-Japan Friendship Hospital, National Center for Respiratory Medicine, Institute of Respiratory Medicine, Chinese Academy of Medical Sciences, National Clinical Research Center for Respiratory Diseases, Beijing, China

**Keywords:** silicosis, proteome, PTM-omics, STAT1, geranylgeranylacetone

## Abstract

Inhalation of crystalline silica dust induces incurable lung damage, silicosis, and pulmonary fibrosis. However, the mechanisms of the lung injury remain poorly understood, with limited therapeutic options aside from lung transplantation. Posttranslational modifications can regulate the function of proteins and play an important role in studying disease mechanisms. To investigate changes in posttranslational modifications of proteins in silicosis, combined quantitative proteome, acetylome, and succinylome analyses were performed with lung tissues from silica-injured and healthy mice using liquid chromatography-mass spectrometry. Combined analysis was applied to the three omics datasets to construct a protein landscape. The acetylation and succinylation of the key transcription factor STAT1 were found to play important roles in the silica-induced pathophysiological changes. Modulating the acetylation level of STAT1 with geranylgeranylacetone effectively inhibited the progression of silicosis. This report revealed a comprehensive landscape of posttranslational modifications in silica-injured mouse and presented a novel therapeutic strategy targeting the posttranslational level for silica-induced lung diseases.

## Highlights

• Conjoint analysis of proteome, acetylome, and succinylome in silicosis.
• Identification of STAT1 acetylation as a therapeutic target for silicosis.
• Validation of the therapeutic role of geranylgeranylacetone on silicosis.

## Graphical Abstract

**Figure:**
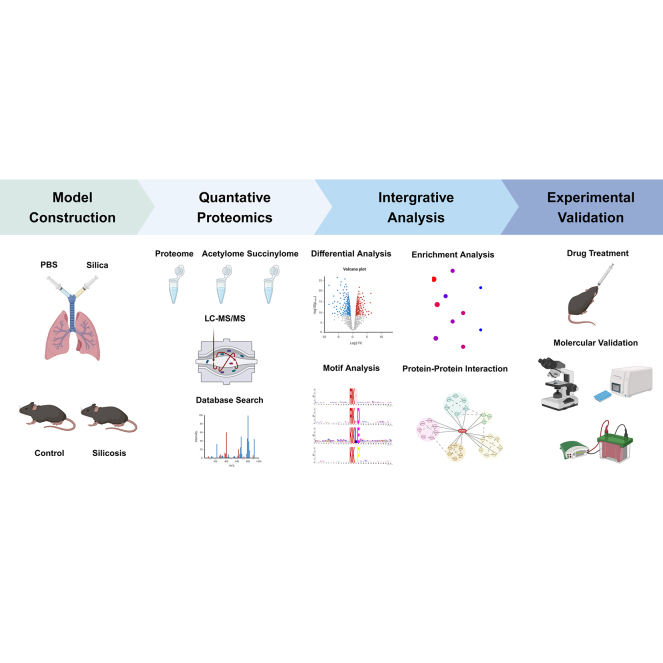

## In Brief

We characterized silicosis-associated changes in protein expression and PTMs (acetylation and succinylation) through quantitative omics analysis, providing a molecular atlas and a systematic overview of this disease. Our results identified the molecular pathways involved in silicosis pathogenesis, along with crucial alteration of PTMs that represented potential drug targets. Furthermore, we confirmed STAT1 acetylation as a novel therapeutic target of silicosis and the results supported the potency of targeting STAT1 acetylation by geranylgeranylacetone in the treatment of silicosis.

Silica exposure can induce severe lung damage or silicosis associated with increased risks of developing cancer, tuberculosis, and chronic obstructive pulmonary disease (1). With the growth of sand blasting industry, more people are at risk of inhaling silica and developing silica-related occupational diseases (2, 3, 4). However, research in silicosis has been mostly restricted to inflammation and fibrosis and do not provide complete understanding of the silica-induced changes in proteins and their functions (5), hindering the development of targeted therapies. Construction of a comprehensive molecular network of silicosis is urgently needed for the exploration of target pathways at a systematic level.

Bulk proteomic datasets can reveal the disease-associated changes in protein expression, allowing proteins with significant roles to be identified in an unbiased manner (6, 7). The first analysis of lungs of silica-exposed mice using iTRAQ yielded 330 differentially expressed proteins compared to healthy controls (8). In cell-based experiments, 196 differentially expressed proteins were reported (fold change ≥1.2) in silica-treated fibroblasts compared with control group (9). In 2020, Yuan *et al.* examined lung tissues of silicosis mice and normal controls and identified 471 differentially expressed proteins between the two groups (10). However, research to date has focused on differentially expressed proteins, with limited mechanistic insight. Compared to traditional proteomics, posttranslational modification (PTM)-omic approaches provide a comprehensive understanding of disease pathogenesis and immunometabolic targets (11). However, these approaches have not been applied to study silicosis yet.

PTM of proteins is an important biochemical process and PTM affects the development of various diseases by regulating the function and degradation of proteins (12, 13, 14). A growing number of PTMs, including phosphorylation, acetylation, succinylation, ubiquitination, SUMOylation, lactylation, have been gradually confirmed. Notably, our recent study revealed the aberrant pathways at protein phosphorylation levels in the lungs of silicosis mice and identified the phosphorylation of EGFR (p-EGFR) and SYK (p-SYK) as potential therapeutic targets in the progression of silicosis (15). Acetylation is a particularly well-studied PTM and succinylation is a recently discovered novel PTM in which metabolically derived succinyl CoA modifies protein lysine groups. Succinylation is a PTM related to multiple biological process, such as cell metabolism, cell proliferation, DNA damage, etc (16). Succinylation is highly correlated with acetylation, and a large number of acetylation at protein sites can also be detected where succinylation occurs (17). Therefore, combined quantitative proteome, acetylome, and succinylome analyses is a preferable strategy to explore the pathogenesis of silicosis. PTM of important transcription factors (TFs) regulates their functions and may determine the expression of large numbers of target genes (18). STAT1, classically regarded as regulator of IFN/STAT signaling in silicosis, can be acetylated and succinylated with significant adjustments on its activity and function (19), while the influence of these PTM alterations in pulmonary fibrotic diseases remains unclear.

Here, we adopted a multi-omic approach to investigate the characteristics of silica-induced lung injury at the protein and PTM levels. We identified changes in protein acetylation and succinylation in silica-injured mouse lungs and then validated the effects of modulating STAT1 acetylation on the extent of inflammation and fibrosis in silica-exposed mice. Our work has revealed important alterations in the lung proteome, acetylome, and succinylome in response to silica injury and identified a novel strategy targeting PTM for the treatment of silicosis.

## Experimental Procedures

### Experimental Design and Statistical Rationale

**Fig. 1:**
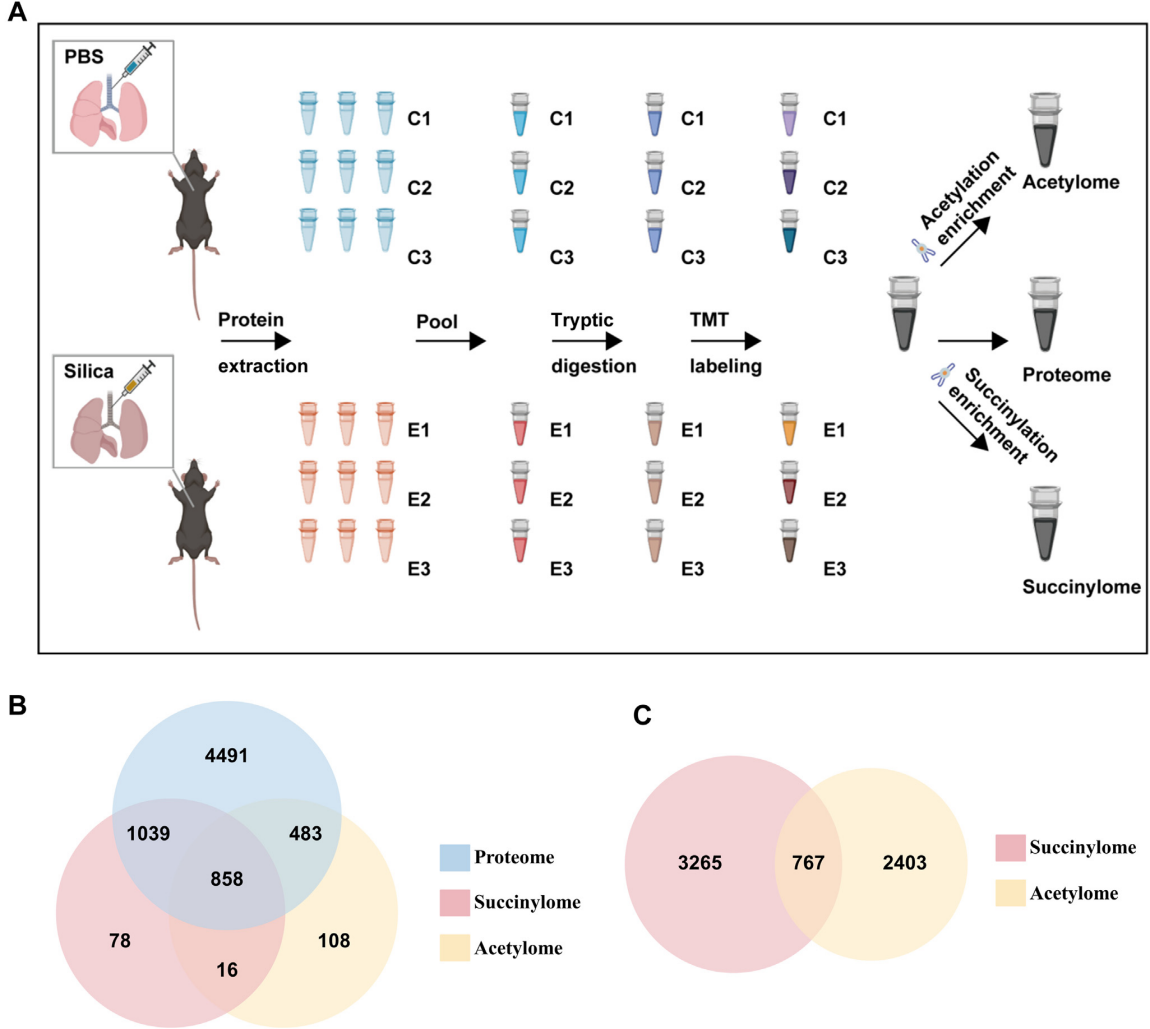
**Altered proteome, acetylome, and succinylome of silicosis mouse lungs.***A*, experimental procedures of multi-omics sequencing. *B*, Venn diagram of quantified proteins in proteome, acetylome, and succinylome. *C*, Venn diagram of acetylated and succinylated amino acid residues for all detected quantified sites in acetylome and succinylome.

To achieve protein, acetylated, and succinylated protein profiling of silica-exposed lungs and discovery of potential therapeutic target, mice (n = 18) were separated into the silicosis group (n = 9) and the control group (n = 9) randomly. For each group, individual samples of lung lobes were collected from nine mice. Each three individual samples from the same group were pooled into a combined sample, therefore six combined samples (n = 3 for control group and n = 3 for silicosis group) in total for analysis. Next, each of the combined sample was divided into three replicates to quantify the proteome, acetylome, and succinylome changes of silica-injured and healthy lungs with tandem mass tags (TMT) (Fig. 1*A*). Then, TMT-labeled combined samples were pooled together in accordance with the experimental purpose. The pooled peptide mixtures of lung tissues from proteomic, acetylomic, and succinylomic samples were pre-fractionated by high pH reverse-phase high-performance liquid chromatography separation into 60 fractions respectively and combined into 24 fractions as to proteome, 4 fractions as to acetylome, and 4 fractions as to succinylome before being subjected to data-dependent acquisition analysis. Additionally, only modified sites with localization probability >0.75 in acetylome and succinylome data were reserved. Differential expressed proteins and modified sites were identified using unpaired *t* test (Benjamini-Hochberg (BH) adjusted *p* < 0.05) and cutoff ratio (silicosis/control) >1.5 or <0.67. Kyoto Encyclopedia of Genes and Genomes (KEGG) and Gene Ontology (GO) enrichment analysis were conducted to confirm the pathways altering significantly (BH-adjusted *p* < 0.01) between silicosis and control groups. Integrative protein-protein interaction network and transcription factor (TF) network were established in Metascape and MetaCore, respectively. Stat1 expression, acetylated, and succinylated modification levels were significantly increased in silica-exposed lungs.

To validate the alteration of Stat1 modification, molecular experiments were performed using the samples mentioned before. To explore the function of Stat1 acetylation in lung fibrosis induced by silica, therapeutic experiments were conducted. The control (n = 18) and silicosis (n = 18) mice were randomized into the PBS group (n = 9 for control group and n = 9 for silicosis group) and the treatment group (n = 9 for control group and n = 9 for silicosis group) with geranylgeranylacetone (GGA). Then, metrics related to lung fibrosis such as inflammatory score, fibrotic score, etc. were measured. The specific statistical method will be narrated in the part below.

### Animal Model Construction and Drug Treatment

Male 6- to 8-week-old C57BL/6J mice (20–25 g) were purchased from Beijing Vital River Laboratory Animal Technology Co, Ltd. All the mice were housed and bred in a specific pathogen-free facility under proper condition (humidity, 60%–70%; temperature, 24–26 °C; 12-h light/dark cycle). The silicosis mouse model was constructed as previously described (20). GGA (GGA) was dissolved in 0.5% carboxymethyl cellulose-Na solvent. After 2-weeks silica/PBS instillation, the mice were given GGA (200 mg/kg) or vehicle in 300 μl solution by daily oral gavage for two consecutive weeks. The animal experiment procedures were approved by the animal care and use committee of Institute of Basic Medicine Sciences, Chinese Academy of Medical Sciences (IBMS, CAMS) with the approval ACUC-A02-2018-008.

### Sample Preparation

The lung lobes from silica-exposed mice and healthy controls were collected and stored in −80 °C until use. Protein extraction and trypsin digestion were performed as previously described (21). Digested peptides were reconstituted in 0.5 M tetraethylammonium bromide and processed according to the manufacturer’s instructions for TMT6-plex labeling (90068, Thermo Fisher Scientific). The peptides from the six samples were mixed respectively with 126-tag, 127-tag, 128-tag, 129-tag, 130-tag, and 131-tag.

TMT-labeled peptides were fractionated by high pH reverse-phase high-performance liquid chromatography with a 300 Extend C18 column (5 μm particles, 4.6 mm inside diameter, and 250 mm length; Agilent). Briefly, peptides were first separated with a gradient of 8% to 32% acetonitrile over 60 min into 60 fractions. For proteomic analysis, peptides were combined into 24 fractions and dried by vacuum centrifugation. For analysis of acetylome and succinylome, peptides were combined into four fractions and vacuum-dried.

To enrich for modified peptides, pre-fractionated peptides dissolved in NETN buffer (100 mM NaCl, 1 mM EDTA, 50 mM Tris-HCl, 0.5% NP-40, pH = 8.0) were incubated with 20 μl pre-washed antibody beads (anti-acetyl-lysine antibody conjugated agarose beads, PTM-104; anti-succinyl-lysine antibody conjugated agarose beads, PTM-402; PTM Biolabs) overnight at 4 °C with gentle shaking. The beads were washed four times with NETN buffer and twice with H_2_O. The bound peptides were eluted from the beads with 0.1% TFA. The supernatant containing modified peptides was collected and lyophilized for liquid chromatography-tandem mass spectrometry (LC-MS/MS) analysis.

### LC-MS/MS Analysis

The peptides were dissolved in 0.1% formic acid (mobile phase A) and loaded onto the EASY-nLC 1000 nanoflow LC system (Thermo Fisher Scientific). Mobile phase B contained 0.1% formic acid and 90% acetonitrile in water. For proteome analysis, the gradient was set as 9% to 80% B in 40 min (400 nl/min). For acetylome or succinylome analyses, the gradient was set as 8% to 80% B in 40 min (500 nl/min).

The nLC system was coupled to a Q Exactive plus mass spectrometer (Thermo Fisher Scientific), acquiring full scans (350–1800 m/z, R = 70,000 at 200 m/z) at a target of 3e6 ions. The instrument was operated in data-dependent acquisition mode. For the MS2 scan, the top 20 precursor ions were selected for fragmentation by higher-energy collision dissociation (target 5e4 ions, maximum injection time 200 ms, isolation window 2.0 m/z, and normalized collision energy 28%) and detected in the Orbitrap (R = 17,500 at 200 m/z).

### Database Searches

Raw MS/MS data were analyzed using the MaxQuant search engine (v.1.5.2.8) (22) against the Swiss-Prot Mouse database (24/06/2019, 17,014 entries) concatenated with the reverse decoy database. The search included variable modifications for oxidized methionine (M) and fixed modification for carbamidomethyl (C) basically. For data analysis of acetylated peptides, additional acetylation on Lys residue and protein N-terminal were specified as variable modifications. For data analysis of succinylated peptides, an additional succinylation on Lys residue was specified as variable modifications. The mass tolerance of precursor ions was set to 20 ppm and the mass tolerance of fragment ions was set to 0.5 Da. The false discovery rate was adjusted to <1% at the protein, peptide, and modification levels. All the raw data have been uploaded to the ProteomeXChange Consortium (https://www.proteomexchange.org/) with PXD identifiers (PXD049146).

### Bioinformatic Analysis

Proteins/peptides in the potential contaminant database and reverse decoy database were excluded. The localization probability of acetylation or succinylation ranged from 0 to 1. Proteins with only identified by sites were filtered. Sites with localization probabilities of >0.75 were selected for further PTM analysis. Only the proteins or sites with at least two or more non-missing values in control or silica-exposed group were kept. Relative abundances of proteins and acetylated and succinylated peptides were quantified using TMT reporter ion intensity from each peptide spectrum match. Both unique and razor peptides were applied for proteome quantitation. Then, the proteome, acetylome, and succinylome data was normalized by median. The missing values were filling with the global minimum value.

Differential proteins and modified sites were identified by *t* test using log2 transformed and median normalized intensity data with BH correction for multiple hypothesis testing to control the false discovery rate. After BH correction, adjusted *p* <0.05 was considered as significant. Upregulated and downregulated proteins/sites were determined as fold change (mean values of silicosis mice/mean values of non-silicosis mice) >1.5 or <0.67, respectively.

Gene set enrichment analysis (GSEA) (23) was conducted using the GSEA software (4.3.2) (https://www.gsea-msigdb.org/gsea/) with default parameters (permutation type = gene_set, number of permutations = 1000, enrichment statistic = weighted, metric for ranking genes = Signal2Noise). The lung fibrosis gene set included *Stn1*, *Dsp*, *Bmp7*, *Calca*, *Cebpb*, *Ccr3*, *Ccr2*, *Csf2*, *Csf3*, *Edn1*, *Egf*, *Eln*, *Fgf1*, *Fgf2*, *Fgf7*, *Ccn2*, *Hgf*, *Hmox1*, *Igf1*, *Il12b*, *Il13*, *Il1b*, *Il4*, *Il5*, *Il6*, *Smad7*, *Cma1*, *Mecp2*, *Mmp2*, *Mmp9*, *Mt2*, *Nfe2l2*, *Pdgfa*, *Pdgfb*, *Plau*, *Ptx3*, *Ccl11*, *Ccl2*, *Ccl3*, *Ccl4*, *Ccl5*, *Cxcl15*, *Cxcl2*, *Sftpa1*, *Sftpc*, *Skil*, *Spp1*, *Tert*, *Tgfa*, *Tgfb1*, *Timp1*, *Tnf*, *Dpp9*, *Elmod2*, *Rtel1*, *Atp11a*, *Fam13a*, *Cysltr2*, *Parn*, *Muc5b* (24).

Stats (version 4.3.1) was used for principal component analysis (PCA) while clusterProfiler (version 4.8.3) was used (25) for KEGG and GO enrichment analysis. The analyses above were conducted using R (version 4.3.1). Motif-x algorithm (soft MoMo, version 5.5.1) was applied to conduct the motif analysis. Integrative protein-protein interaction analysis and related function annotation were performed using Metascape (version 3.5) (26). TF-network was established in MetaCore.

### Histological Staining and Analysis

H&E staining and Masson staining were conducted for each individual sample with three replicates according to the instructions of corresponding kit (Hematoxylin-Eosin Stain Kit, Solarbio, G1120; Masson's Trichrome Stain Kit, Servicebio, G1006). The experimental steps were followed by the manufacturer’s protocol. Chronic inflammation of lungs was evaluated using the inflammatory score narrated before (27) based on the H&E staining. Masson staining aimed to analyze the levels of collagen and evaluate the degree of pulmonary fibrosis using the fibrotic score defined by modified Ashcroft scale (28). The mean values of inflammatory scores and fibrotic scores for all evaluated images were calculated on the basis of the tissue sections with complete lung lobes.

For immunohistochemistry, paraffin tissue sections were heated at 65 °C in the oven for 30 min then soaked into xylene and alcohol with gradient concentration for 5 min in each dewax solution. After washing with PBS, the tissue sections were treated with citrate buffer (pH 6.0), exposed to heat-induced epitope retrieval at 100 °C for 20 min, and washed with PBS. The tissue sections were treated with 3% hydrogen peroxide for 10 min, 0.3% Triton-100 for 10 min, and goat serum for 30 min at room temperature consecutively. PBS was applied to wash the tissue section after each treatment. Then the tissue sections were incubated with primary antibodies against anti-Collagen I (Abcam, ab254113, 1:200) at 4 °C overnight. After washing with PBS, the secondary antibodies (Goat anti-Rabbit IgG, Origene, PV-6001) were subsequently added on the tissue sections to incubate at room temperature for 60 min. After washing with PBS, these sections were stained using 3,3-diaminobenzidine (Origene, ZLI-9018) until turning brown and then washed with PBS. Subsequently, all the tissue sections were counterstained with hematoxylin and then washed with PBS. Finally, these tissue sections were dehydrated with ethanol and toluene, then sealed with neutral gum. Photographs were taken under microscope and the percentage of positive area was taken to be statistical basis as per the tissue sections with complete lung lobes.

### Quantitative Real-time PCR

Total RNA was extracted from mouse lung lobes with TRIzol regent (Invitrogen). The concentration of total RNA was quantified by the optical density at 260 nm according to the manufacturer’s instructions. Then, complementary DNA (cDNA) was generated from 1 μg of total RNA with a cDNA reverse transcription kit (KR103, Tiangen Biotechnology). The amplification and detection of related cDNA were conducted with the SYBR Green I Q-PCR kit (TransGen Biotech) using a Bio-Rad IQ5 system (Bio-Rad). The corresponding primer sequences are listed in supplemental Table S1. The relative level of mRNA was calculated using the 2^−ΔΔCt^ method (29) and normalized to β-actin mRNA expression level.

### Immunoprecipitation and Western Blotting

Mouse lung tissue samples were lysed in RIPA buffer (50 mM Tris–HCl (pH 7.4), 150 mM NaCl, 1% Triton X-100, 1 mM EDTA, 10% glycerol) with protease/phosphatase inhibitor cocktail (5872S, Cell Signaling Technology, 1:1000), 0.3 mM DTT, 5 mM nicotinamide, and 1 mM sodium butyrate. Total 500 μg of protein were used for immunoprecipitation with indicated dilution of each antibody (anti-STAT1, 14994, Cell Signaling Technology, 1:100).

Western blotting was performed according to standard protocols. The antibodies and dilutions were anti-β-actin (sc-5286, Santa Cruz, 1:10,000), anti-STAT1 (14994, Cell Signaling Technology, 1:1000), anti-acetyl-lysine (PTM-105, PTM Bio, 1:1000), and anti-succinyl-lysine (PTM-401, PTM Bio, 1:1000).

### Statistical Analysis

The quantitative data was tested using Graphpad Prism 9.0.0 (GraphPad Software, Inc, https://www.graphpad.com/). The Shapiro–Wilk or Kolmogorov–Smirnov test was utilized to test the normally distribution of the data and Levene test to test the equality of variance. Normally distributed data with equal variances were analyzed using Student *t* test or one- or two-way ANOVA, and non-normally distributed data or data with unequal variances were analyzed using Mann–Whitney U test or Kruskal–Wallis test. In addition, post hoc comparisons for multiple groups were performed using Student *t* test or Mann-Whitney U test followed by Bonferroni correction. If not specified, *p* value less than 0.05 was regarded as statistically significant.

### Illustrations and Figures

The graphic abstract and workflow were created from the templates in BioRender (https://www.biorender.com/). The heatmap, volcano plot, PCA, and enrichment analysis visualization were depicted in R (version 4.3.1). Pheatmap (version 1.0.12) was used for the heatmap while ggplot (version 3.5.0) was used for the volcano plot. PCA visualization was processed using ggbiplot (version 0.55) while the enrichment analysis visualization was processed using clusterProfiler (version 4.8.3). The quantitative data was visualized using Graphpad Prism 9.0.0 (GraphPad Software, Inc).

## Results

### Altered Proteome, Acetylome, and Succinylome in the Lung Tissue of Silicosis Mouse Model

**Supplemental Figure S1:**
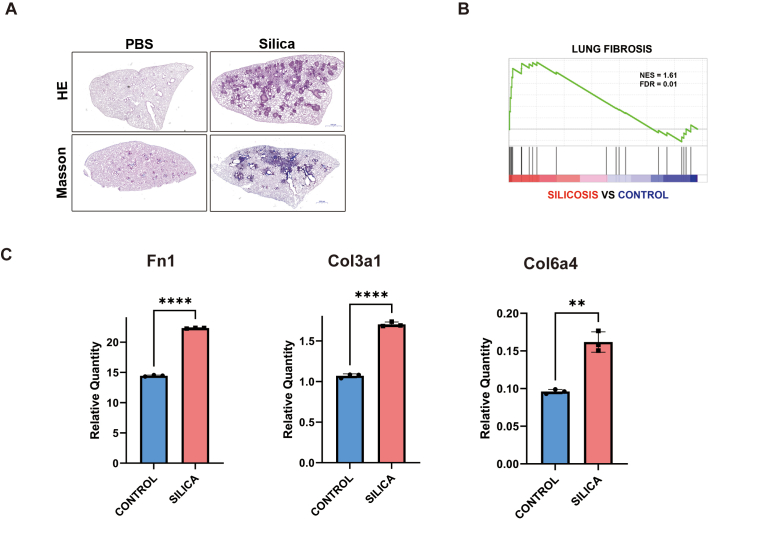


**Supplemental Figure S2:**
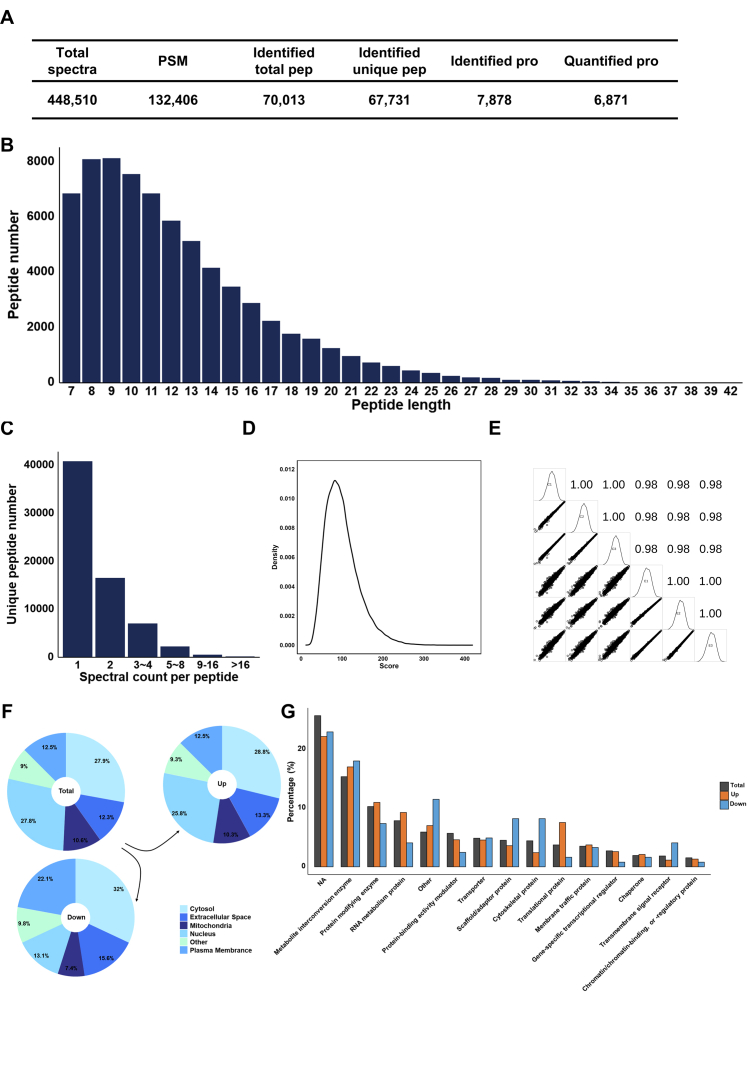


The right middle lung lobes from silica-exposed mice and healthy controls were collected. H&E and Masson staining indicated the model was made successfully (supplemental Fig. S1*A*). For each experimental group, samples were collected from nine mice and pooled into three independent samples for analysis. Next, each sample were divided into three replicates to quantify the proteome, acetylome, and succinylome changes of silica-injured and healthy lobes with TMT (Fig. 1*A*). We separated the labeled peptides into multiple fractions with high-performance liquid chromatography, then combined the fractioned labeled peptides. As for acetylome and succinylome samples, we enriched them with anti-Ac and anti-Sc antibodies respectively before conducting LC-MS/MS analysis. After that, the LC-MS/MS-isolated lysine-acetylated/succinylated peptides were searched against the Swiss-Prot Mouse database and then normalized. In total, 6871 proteins, 3170 acetylated sites from 1465 proteins, and 4032 succinylated sites from 1991 proteins were quantified in the analyses of the proteome (supplemental Fig. S2, and supplemental Tables S2–S5), acetylome (supplemental Table S4) and succinylome (supplemental Table S5). Importantly, 858 proteins were shared among the proteomic, acetylomic, and succinylomic data (Figs. 1*B*), and 767 lysine residues were both acetylated and succinylated (Fig. 1*C*).

### Global Analysis of Proteome in Silica-Injured Mice Lungs

**Fig. 2:**
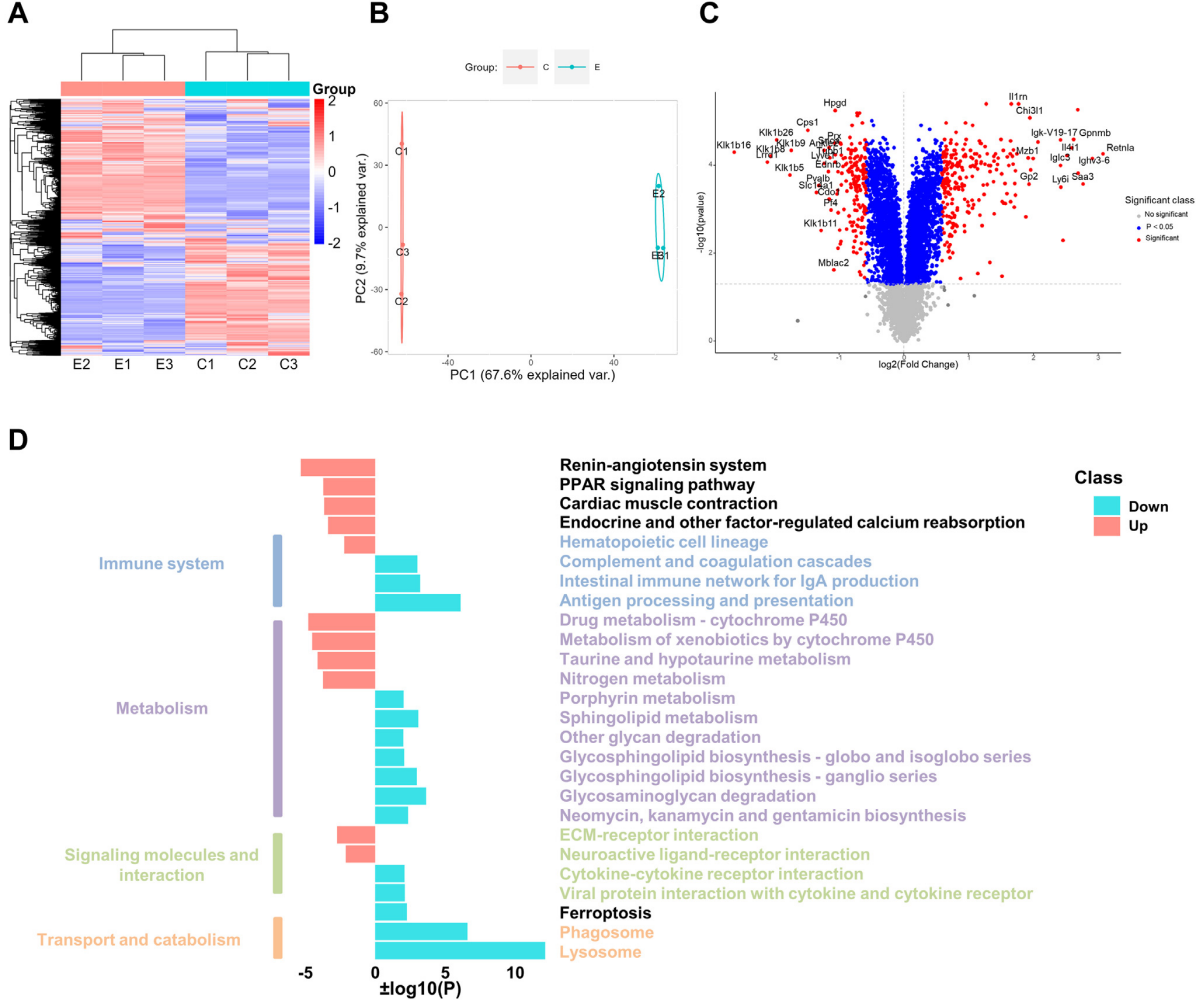
**TMT quantitative proteome landscape of silicosis mouse lungs.***A*, hierarchical clustering of TMT quantitative proteome data. *B*, principal component analysis (PCA) of TMT quantitative proteome data. The *red dots* represent lung samples from healthy mice and the *green dots* indicate lung samples from silicosis mice. *C*, volcano plots to show the altered proteins between lungs from control subjects with silicosis. The *red dots* represent proteins, which meet the criteria of *p* < 0.05 and fold change >1.5 or <0.67, while the *blue dots* represent proteins, which meet the criteria of *p* < 0.05. Top 20 upregulated and downregulated proteins (ordered by Log2 FC) were labeled with gene name if could be mapped to. *D*, a summary of dysregulation functions in silicosis based on KEGG enrichment analysis (*p* < 0.01). Pathways mapped to “Human Diseases” were not shown. KEGG, Kyoto Encyclopedia of Genes and Genomes; TMT, tandem mass tag.

To evaluate the changes of protein expression and identify independent clusters in the silicosis group compared to the control group, two independent analyses, namely an unsupervised hierarchical clustering analysis (Fig. 2*A*) and PCA (Fig. 2*B*), were carried out. A total of 422 differently expressed proteins were identified (Fig. 2*C*, top 20 in supplemental Table S6, adjusted *p* < 0.05 and Silicosis/Control >1.5 or <0.67), of which 243 were upregulated and 179 were downregulated. GSEA representing lung fibrosis showed promotion in silica-exposed mice (supplemental Fig. S1*B*). Fibrotic markers were also upregulated in lungs from silica-exposed mice (supplemental Fig. S1*C*). Furthermore, based on KEGG pathway analysis (*p* < 0.01), lungs from silica-induced mice showed enrichment of pathways involved on “Immune system,” “Metabolism,” “Signaling molecules and interaction,” and “Transport and catabolism” (Fig. 2*D*).

### Large Scale Profiling of Acetylation Sites in Mice Lung

**Fig. 3:**
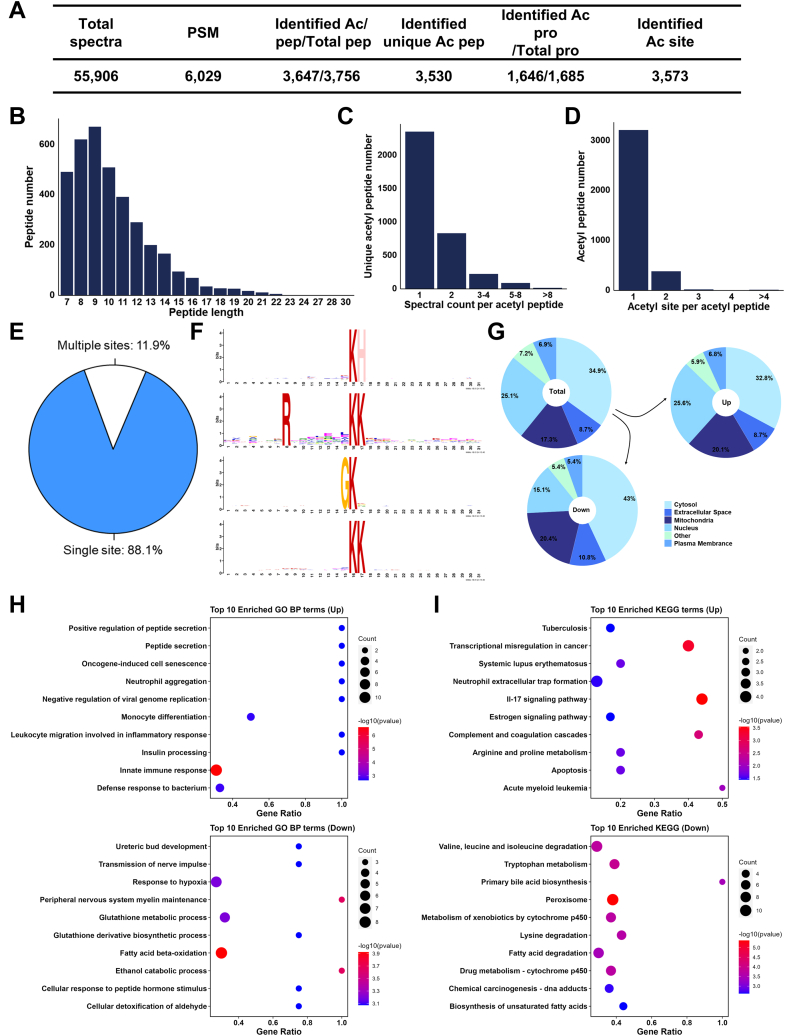
**Large scale profiling of acetylation sites in silica-injured mouse lungs.***A*, identified spectra and peptide for TMT-labeled acetylated proteins. *B*, distribution of acetylated peptides in peptides of different length. *C*, distribution of unique acetylation peptides number on the spectral count of each acetylated peptides. *D*, distribution of acetylated peptides number in the numbers of acetylation sites. *E*, pie chart of modification type distribution of acetylation sites. *F*, motif analysis of acetylation sites in detected mouse lungs. *G*, subcellular localization enrichment of quantified acetylated proteins. *H*, GO BP analysis in differentially acetylated proteins (top 10). *I*, enrichment analysis of KEGG pathways in differentially acetylated proteins (top 10). GO, gene ontology; KEGG, Kyoto Encyclopedia of Genes and Genomes; TMT, tandem mass tag.

**Supplemental Figure S3:**
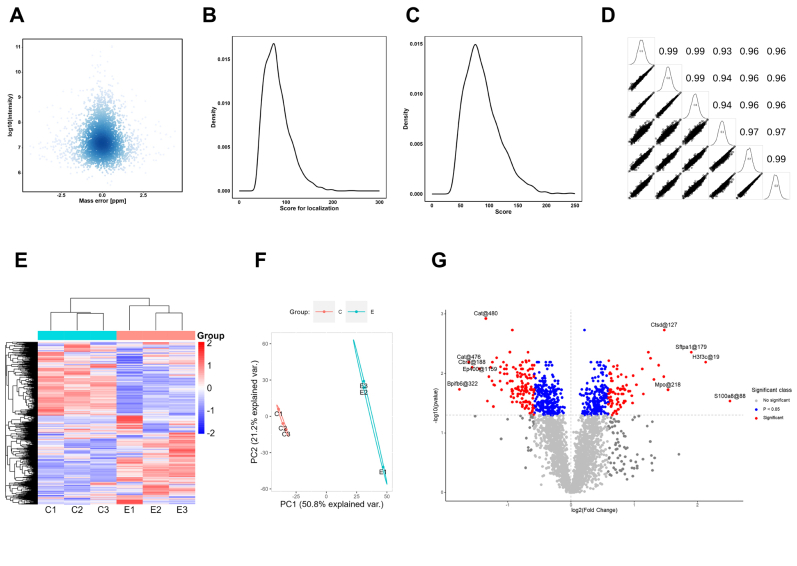


Taken together, we totally identified 3573 unique acetylated sites from 1646 acetylated proteins (Fig. 3*A*, and supplemental Fig. S3). Interestingly, acetylation was not uniformly distributed in the peptides of different lengths (Fig. 3, *B* and *D*). Despite the majority of identified peptides had a single acetylation site (88.1%, 3213/3647), there were also 434 peptides which included at least two acetylated sites (Fig. 3*E*).

Then we evaluated the sequence preferences of the lysine acetylation in silica-injured mice lungs with Motif-x, which can be applied to search for putative acetylation motifs in the identified acetylation dataset. Specific motifs significantly enriched in our identified acetylation dataset were identified, including Kac-H, R-X-X-X-X-X-X-X-Kac-K, G-Kac, and Kac-K, where Kac represents the acetylated residue and X represents any amino acid residue (Fig. 3*F*). And the upregulated acetylated proteins were shown more in mitochondria compared with the total quantified acetylated proteins, while the downregulated were shown more in cytosol (Fig. 3*G*).

To better understand the biological function of acetylation *in silica* injuries, we annotated the significantly changes in acetylated proteins (Fold change >1.5) through GO and KEGG enrichment analysis. GO annotations of upregulated proteins were significantly enriched in innate immune response (*p* = 2.47E-7) and defense response to bacterium (*p* = 1.64E-3) (Fig. 3*H*), while those of downregulated ones were significantly enriched in fatty acid beta-oxidation (*p* = 1.20E-4) and ethanol catabolic process (*p* = 2.21E-4). Furthermore, the KEGG pathways of up-regulated proteins were significantly enriched in IL-17 signaling pathway (*p* = 2.85E-04) and transcriptional misregulation in cancer (*p* = 4.61E-04) (Fig. 3*I*), while the pathways of downregulated proteins were significantly enriched in peroxisome (*p* = 4.25E-06) and tryptophan metabolism (*p* = 1.29E-04).

### Large Scale Profiling of Succinylation Sites in Mice Lung

**Fig. 4:**
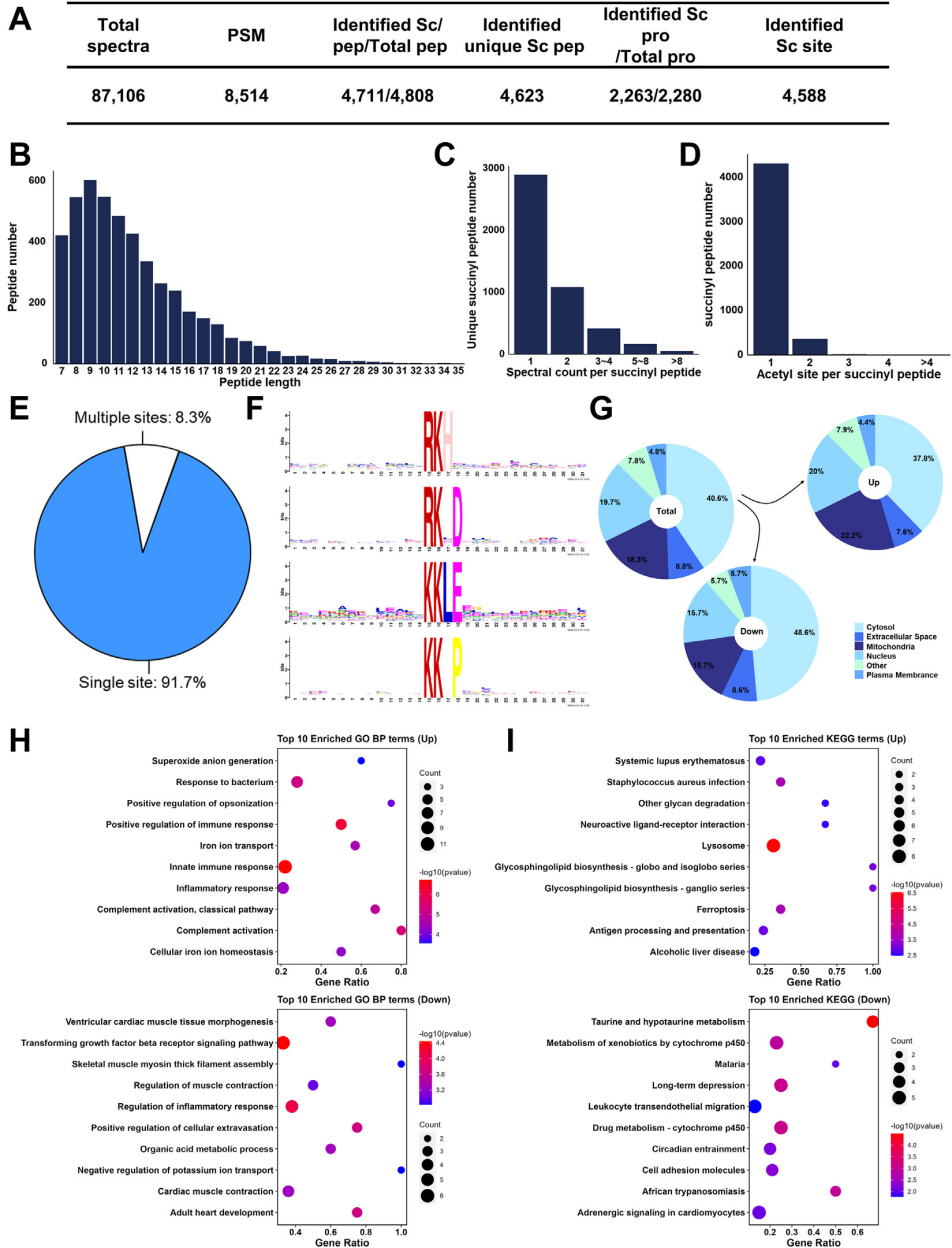
**Large scale profiling of succinylation sites in silica-injured mouse lungs.***A*, identified spectra and peptide for TMT-labeled succinylated proteins. *B*, distribution of succinylated peptides in the peptides of different length. *C*, distribution of unique succinylation peptides number on the spectral count of each succinylated peptides. *D*, distribution of succinylated peptides number in the numbers of succinylation sites. *E*, pie chart of modification type distribution of succinylation sites. *F*, motif analysis of succinylation sites in detected mouse lungs. *G*, subcellular localization enrichment of succinylated proteins. *H*, GO BP analysis in differentially succinylated proteins (top 10). *I*, enrichment analysis of KEGG pathways in differentially succinylated proteins (top 10). GO, gene ontology; KEGG, Kyoto Encyclopedia of Genes and Genomes; TMT, tandem mass tag.

To acquire the detailed landscape of lysine succinylation events in silica-injured lung lobes, we applied succinylome and then identified a total of 4588 unique succinylated sites from 2263 succinylated proteins (Fig. 4*A*, and supplemental Fig. S4). Coincidentally, succinylation was also not uniformly distributed in the peptides of different lengths (Fig. 4, *B*–*D*) and despite the majority of identified peptides had a single succinylated site (91.7%, 4320/4711), there were 391 peptides which included at least two succinylated sites (Fig. 4*E*).

After that, to evaluate the sequence preferences of the lysine succinylation in mice lungs, we used Motif-x, then identified four specific motifs significantly enriched in our identified succinylation dataset, including R-Ksc-H, R-Ksc-X-D, K-Ksc-L-E, K-Ksc-X-P (Fig. 4*F*). And the upregulated succinylated proteins were shown more in mitochondria compared with the total quantified succinylated proteins, while the downregulated were shown more in cytosol (Fig. 4*G*).

Also, we applied GO and KEGG enrichment analysis to reveal the significant processes participated by differentially succinylated proteins. Innate immune response as well as transforming growth factor beta receptor signaling pathway were significantly identified in GO Biological Process (Fig. 4*H*), while lysosome and taurine and hypotaurine metabolism were highlighted in KEGG pathway enrichment results (Fig. 4*I*).

### Integrated Functional Analysis Revealed the Key Modulator Stat1 in Silicosis

**Fig. 5:**
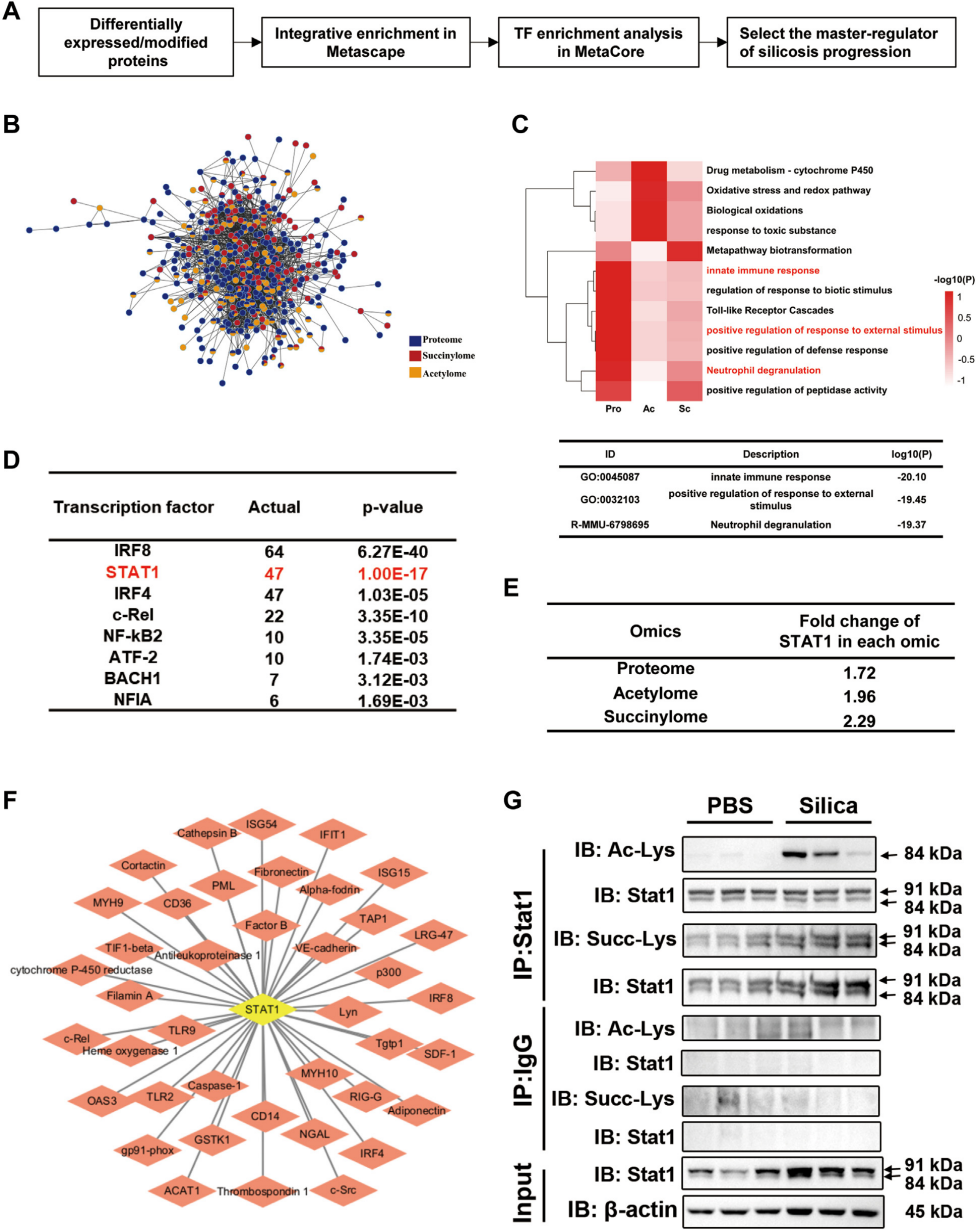
**Validation of significantly changed proteins in silicosis mice.***A*, schematic diagram of master regulator exploration in silicosis mice. *B*, enrichment protein–protein interaction (PPI) network colored by counts in each ‘-omics’ data (full connection). *Blue* represents proteome, *red* represents acetylome, and *orange* represents succinylome. *C*, heatmap of enriched terms across input gene lists, colored by *p* values. *D*, over connection ranks of differentially expressed, acetylated, or succinylated proteins (top 4). *E*, the fold change of Stat1 in each ‘-omics’ data. *F*, the significant interactions with Stat1 shown as network. *G*, Co-IP data of Stat1.

The integrated analysis was carried on (Fig. 5*A*). We made a pairwise comparison among the intensity of proteins, acetylated sites, and succinylated sites to calculate the Pearson correlation coefficients among the protein interaction pairs obtained through Metascape analysis. We found complex correlations (Pearson correlation coefficient >0.6 or <0.6, *p* < 0.05) among these pairs (2353/2380) (Fig. 5*B*). Integrated functional enrichment analysis with Metascape showed that the pathways common to proteome, acetylome, and succinylome included the innate immune response, positive regulation of response to external stimulus, and neutrophil degranulation (Fig. 5*C*).

In order to narrow down the pathogenic changes associated with silicosis to differentially expressed and modified proteins, TF-network analysis was performed in Metacore to identify the key modulators in silicosis. Most of the integrated pathways were regulated by key transcription factors (Irf8, Stat1, and Irf4) (Fig. 5, *D*–*F*). Co-immunoprecipitation was carried out to investigate Stat1, which may play a significant role in silicosis mechanisms. The IP-IB analysis revealed significantly increased acetylation in Stat1β and increased succinylation in Stat1α and Stat1β (Fig. 5*G*), which enriched our understanding of modifications in Stat1 in silicosis beyond proteome analysis results.

### GGA Treatment Ameliorated Silica-Induced Lung Inflammation and Fibrosis through Modulating Stat1

**Fig. 6:**
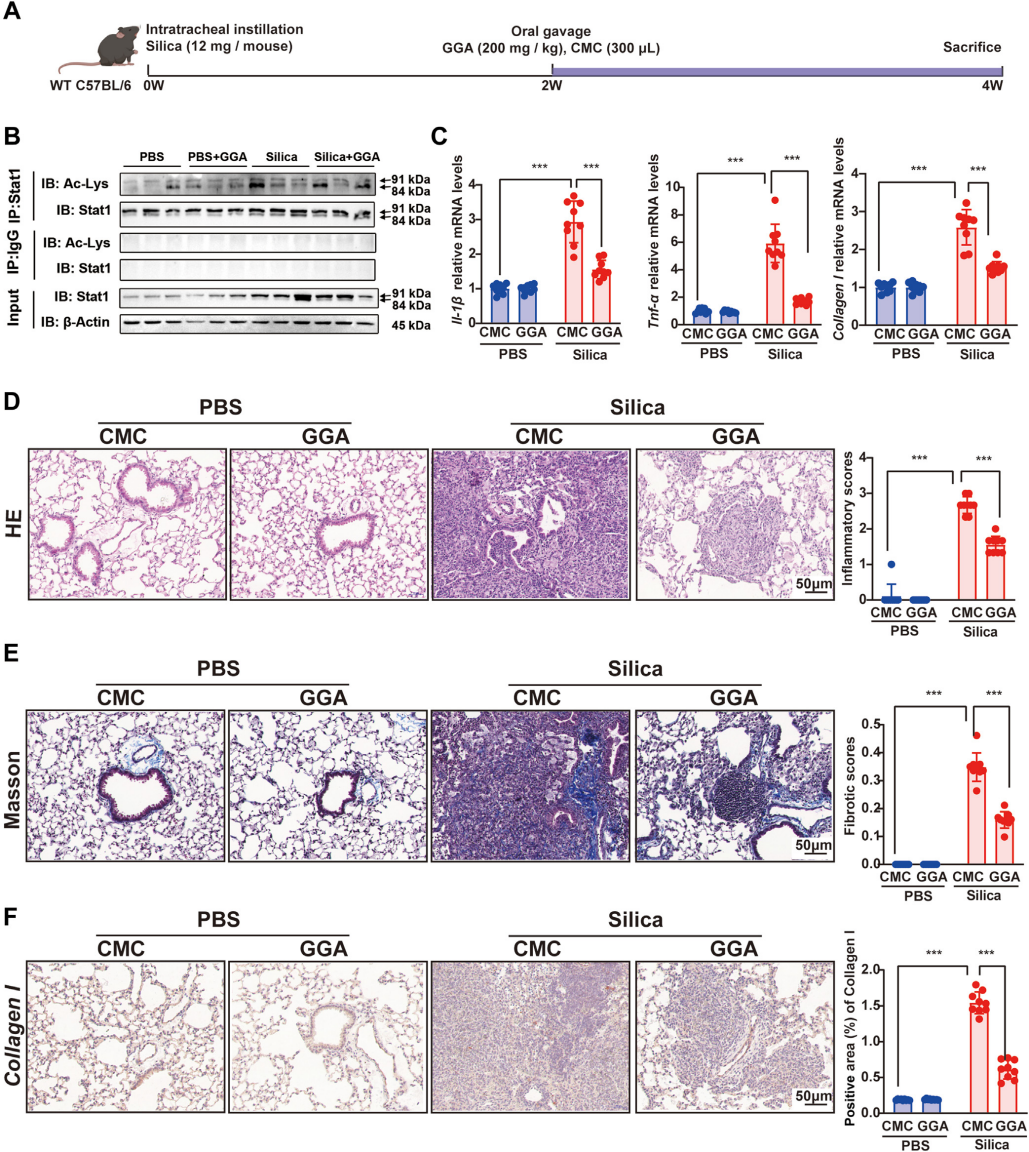
**GGA treatment suppress pulmonary dysfunction, inflammation, and fibrosis in silicosis mice.***A*, schematic diagram of master regulator exploration in silicosis mice. *B*, Co-IP data of Stat1. *C*, the relative mRNA levels of *Il-1β*, *Tnf-α*, and *Collagen I* in lung tissues. Representative images of (*D*) H&E and (*E*) Masson staining, and the lower scale bar indicates 50 μm. Quantification of pulmonary inflammation and fibrosis, n = 9 each group in (*C*–*E*). *F*, the statistical analysis results of positive area of Collagen I immunoprecipitation. (PBS group: n = 9 each group; silica group: n = 9 each group). All the above experiments were performed at least three times. All the quantitative results are presented as mean ± SD. All experiment groups were compared by a two-way ANOVA followed by Bonferroni’ s multiple comparisons test, ns: no significance; ∗∗∗*p* < 0.001.) GGA, geranylgeranylacetone; PBS, phosphate-buffered saline.

To verify whether acetylation of Stat1 plays as a potential drug target in silica-induced pulmonary fibrosis, we performed GGA administration in this study. GGA, an innocuous Hsp70 inducer, exhibits anti-inflammatory properties and effectively mitigates the progression of pulmonary fibrosis. Hsp70 can regulate the activity or stability of Stat1 by interacting with it, thereby influencing the signal transduction and transcriptional regulation mediated by Stat1. Silica-exposed mice and controls were divided into two groups and then given GGA (200 mg/kg) or sodium carboxymethyl cellulose vehicle daily for 2 weeks (Fig. 6*A*). We observed that the level of acetylation of Stat1 was increased (Fig. 6*B*) after GGA treatment. The mRNA levels of *Il-1β* and *Tnf-α* as well as H&E staining revealed that GGA attenuated lung inflammation in the mouse model of silicosis (Fig. 6, *C* and *D*). Meanwhile, qPCR and immunohistochemistry results of collagen I as well as Masson's trichrome staining revealed that GGA effectively reduced the indicators of fibrosis in silica-exposed mice (Fig. 6, *C*–*F*). Taken together, these data indicate that GGA can attenuate the progression of silicosis in mice through interfering posttranslational acetylation of Stat1.

## Discussion

To date, research has mainly focused on clinical features of silicosis (30, 31), while the molecular mechanisms of disease pathogenesis remain unclear. To our knowledge, this is the first study applying TMT proteomics and PTM-omics to construct a systematic network, allowing the modulation of acetylation to be explored as a potential treatment for silicosis.

TMT-based proteomics provides higher throughput, sensitivity, precision, and reproducibility than other types of quantitative methods. According to the Center for Strategic Scientific Initiatives, TMT proteome enables innovative biological insight formation (32, 33, 34). In this case, applying TMT proteomics to compare the lungs from silicosis mice with healthy controls, a large number of differentially expressed proteins were identified, which were more than previous reports using other methods and with highly improved detection precision.

Here, we revealed the signaling network of silicosis pathogenesis at protein and PTM levels. We identified the key biomarkers of known mechanisms in silicosis, such as immune system, signaling molecules and interaction, and transport and catabolism. We also revealed interesting processes in silicosis pathogenesis, such as metabolism dysregulation. Metabolic dysregulation is known to promote the chronic symptoms and cardiovascular complications in silicosis (35, 36, 37).

To the best of our knowledge, this is the first characterization of acetylome and succinylome in silicosis. We focused on the PTM changes in transcription factors, including STAT1, IRF8, IRF4, c-Rel, and NF-kB2.The dynamics of PTMs regulation in key TFs need to be further explored to comprehensively understand the mechanism of silicosis, but we definitely provide important candidates for deep studies. As STAT1 was the most significantly changed member among differentially modified TFs (38, 39), it was selected as a target for therapeutic experiments.

STAT1 is known to participate in the protective responses to injury and is responsible for activating the transcription of key genes involved in cell survival, growth regulation, apoptosis, and differentiation. In previous studies, STAT1 was regarded as an IFN-γ TF driving Th1 cell differentiation and reducing fibrosis *via* interferon signaling and TGF-β1 pathways. Although there is no published work of STAT1 in silicosis, the study of STAT1 in pulmonary fibrosis is well described. M2 macrophages can carry miR-129-5p to pulmonary interstitial fibroblasts and inhibit STAT1 gene expression, which may lead to the proliferation of fibroblasts and promote pulmonary fibrosis. The downregulation of miR-129-5p can significantly promote STAT1 gene expression in macrophages to inhibit pulmonary fibrosis in rats (40). In bleomycin-induced pulmonary fibrosis mice model, HDAC3 promoted EMT, inflammation, and pulmonary fibrosis development by activating STAT1 signaling. A combination therapy of Tanshinone IIA and Puerarin for IPF was proposed to alleviate IPF, and IL-6-JAK2-STAT3/STAT1 is the key mechanism of the combination therapy. In a bleomycin-induced pulmonary fibrosis mice model, IL-35 activated STAT1 and STAT4, which in turn suppressed DNA enrichment of STAT3 and inhibited the fibrosis process (41).

Consistently, antisense oligonucleotides against STAT1 could inhibit the secretion of TNF-α, IL-8, and NO by alveolar microphages in bleomycin-induced inflammation (42). Given these key functions of STAT1 in inflammation and fibrosis, it is highly important to correct the dysregulated biological activity of this TF. As shown in previous research, acetylation of STAT1 (modulated by the HDAC family) can regulate NF-kB activity, apoptosis, and the transcription of inflammatory genes. Modulating STAT1 acetylation represents a promising strategy for the treatment of pulmonary fibrosis, as shown by the results of our present study.

GGA acts as an inducer of HSP70 and regulates the activity of STAT1 mainly through HSP70. GGA was reported to accelerate tissue remodeling by suppressing deacetylase activity. Studies have reported beneficial effects of GGA in bleomycin-induced lung fibrosis, which was attributed to its actions in modulating TGF-β1–dependent fibroblast activation, accumulation of inflammatory cells, and secretion of pro-inflammatory cytokines (43, 44). In this case, we hypothesized that GGA could suppress silica-injured inflammation and fibrosis progression by modulating acetylation. Our experiment results showed that GGA treatment increased the acetylation of STAT1 and effectively reduced pulmonary inflammation and fibrosis. These findings suggest that GGA may be tested clinically for drug/particle-induced pulmonary fibrosis in future studies.

## Data Availability

All materials displayed in this paper are available. The raw MS data can be accessed *via* ProteomeXchange with the identifier PXD049146. The annotated spectra have been deposited *via* MS-Viewer (https://msviewer.ucsf.edu/prospector/cgi-bin/msform.cgi?form=msviewer) with following search keys: nddriwrnmd for proteome data, qnhorhszn1 for acetylome data, and r4mvsz8pfc for succinylome data.

## Supplemental Data

This article contains supplemental data.

## Conflicts of interest

All authors declare no competing interests.

## Figures and Tables

**Supplemental Figure S4:**