# Supplementary material for: Proteome, Lysine Acetylome, and Succinylome Identify Posttranslational Modification of STAT1 as a Novel Drug Target in Silicosis
Source: Mol Cell Proteomics. 2024 Apr 17;23(6):100770. doi: 10.1016/j.mcpro.2024.100770 (PMC11107463; doi:10.1016/j.mcpro.2024.100770)
Supplement: Supplemental Figure Legends [file mmc2.docx]

**Figure S1 Quality Control of Lung Tissue from Silica-Exposed Mice and Healthy Controls**

**A)** Representative images of HE (hematoxylin-eosin) and Masson of silica-exposed and healthy mice. **B)** GSEA (gene set enrichment analysis) of proteome of silica-exposed and healthy mice representing lung fibrosis. NES, normalized enrichment score. FDR, false discovery rate. **C)** Expressions of fibrotic markers in proteome. All the quantitative results are presented as mean ± SD.

**Figure S2 Quality Control of LC-MS/MS in TMT Quantitative Proteome**

**A)** Identified spectra and peptide and TMT labelled quantitative proteins. **B)** Peptide length distribution for all TMT labelled identified proteins. **C)** Histogram of peptide-to-spectrum matches (PSMs) per unique identified peptide. **D)** Andromeda score distribution for all identified peptides. **E)** Scatter plot and density distribution based on log2(intensity) of quantified proteins. Pearson correlation coefficients were calculated and displayed in the diagram. **F)** Location and **G)** classification analysis of all the proteins identified in TMT quantitative proteome.

**Figure S3 Quality Control of LC-MS/MS in TMT Quantitative Proteome with Acetylation Enrichment**

**A)** Mass error distribution of all acetylated peptide. **B)** Localization score distribution for all acetylated peptides. **C)** Andromeda score distribution for all acetylated peptides. **D)** Andromeda score distribution for all acetylated proteins. **E)** Hierarchical clustering of TMT quantitative acetylome data. F) Principal component analysis (PCA) of TMT quantitative acetylome data. The red dots represent lung samples from healthy mice and the green dots indicate lung samples from silicosis mice. **G)** Volcano plots of the -log10 *P* value vs. the log2 Fold Change comparisons between lungs from silicosis subjects with control. Top 5 up-regulated and down-regulated sites (ordered by Log2 FC) were labeled with gene name if could be mapped to.

**Figure S4 Quality Control of LC-MS/MS in TMT Quantitative Proteome with Succinylation Enrichment**

**A)** Mass error distribution of all succinylated peptide. **B)** Localization score distribution for all succinylated peptides. **C)** Andromeda score distribution for all succinylated peptides. **D)** Andromeda score distribution for all acetylated proteins. **E)** Hierarchical clustering of TMT quantitative succinylome data. **F)** Principal component analysis (PCA) of TMT quantitative succinylome data. The red dots represent lung samples from healthy mice and the green dots indicate lung samples from silicosis mice. **G)** Volcano plots of the -log10 *P* value vs. the log2 Fold Change comparisons between lungs from silicosis subjects with control. Top 5 up-regulated and down-regulated sites (ordered by log2 FC) were labeled with gene name if could be mapped to.
